# Small-sided games in floorball: impact on technical actions in players aged 7–18

**DOI:** 10.1186/s13102-026-01992-3

**Published:** 2026-08-15

**Authors:** Mårten Storm, Taru Tervo, Jonas L. Markström

**Affiliations:** 1 Swedish Floorball Federation, Solna, Sweden; 2 Floorball Research and Development Centre, Umeå University, Umeå, Sweden; 3 Umeå School of Sport Sciences, Umeå University, Umeå, Sweden; 4 Department of Community Medicine and Rehabilitation, Unit of Physiotherapy, Umeå University, Umeå, Sweden

**Keywords:** Small-sided games, Floorball, Youth athletes, Technical actions, Team sports

## Abstract

**Background:**

Small-sided games (SSGs; modified match play with fewer players and a smaller pitch) are widely used in team sports to develop adaptable movement skills and decision-making in game-like contexts. However, evidence is limited on how different SSG formats affect technical actions in youth floorball. This study examined how different SSG formats influence technical actions by outfield players in floorball, and whether these effects differ by age group and biological sex.

**Methods:**

Twenty-four youth floorball teams across four age groups, with equal numbers of male and female teams, participated in this observational study. Teams performed four SSG formats (2v2, 3v3, 4v4, and 5v5) in a randomized order while being video-recorded. Technical actions by outfield players were analyzed using multilevel linear models to examine the effects of SSG format, age group, and sex.

**Results:**

SSG format was a significant predictor of player involvement across all outcomes (*p* < 0.001), with smaller formats associated with greater individual engagement. Age group influenced several technical actions, whereas sex showed no significant effects. Significant age group*SSG interactions indicated that the effects of SSG format varied across age groups.

**Conclusions:**

These findings provide sport-specific evidence for youth floorball and show that SSG effects on technical involvement vary with age. Coaches should therefore consider age when selecting SSG formats, as smaller formats increase player engagement while larger formats may better support tactical involvement in older players.

**Supplementary Information:**

The online version contains supplementary material available at 10.1186/s13102-026-01992-3.

## Introduction

In team sports, the ability to adapt technical actions to dynamic and unpredictable game contexts is a key determinant of performance [1]. Rather than relying on isolated or rehearsed movements, players must continuously perceive and act on relevant information to achieve goal-directed actions in real time. These responses are typically expressed through technical actions such as passing, dribbling, and shooting, executed under varying tactical conditions. Consequently, coaches increasingly design training environments that emphasize variability, decision-making, and tactical awareness to promote transfer to gameplay. To implement these principles in practice, coaches frequently use small-sided games (SSGs), which are modified versions of formal match play that reduce the number of players and the size of the playing area.

These game-based formats have gained popularity in sports like soccer, futsal, rugby, basketball, and field hockey as they offer greater involvement per player, promote more frequent technical actions, and are considered to simulate real-game conditions more effectively than traditional drills [2–5]. The theoretical foundation for this approach is rooted in nonlinear pedagogy, drawing on ecological dynamics and dynamic systems theory. This framework conceptualizes skill acquisition as emerging from the interaction among the athlete, the task, and the environment [6, 7]. Rather than prescribing a specific technique, nonlinear pedagogy emphasizes exploratory learning through representative game scenarios. By adjusting task constraints such as the number of players, available playing area, or rules, coaches can create affordances that support the development of tactical understanding, decision-making, and technical proficiency [8]. Thus, the analysis of technical actions provides a practical indicator of player engagement and skill expression in representative contexts.

In team sports, SSG with fewer players generally results in more technical actions per player. For example, studies in field hockey, basketball, and soccer have reported increases in ball involvements, passes, and decision-making actions as player numbers are reduced [5, 9, 10]. In SSGs with larger team sizes, older youths tend to perform more technical actions than younger players, likely because they can better exploit the playing area due to greater physical maturity [9, 10].

Sex may also influence responses to task constraints, as boys and girls follow different maturation trajectories during adolescence, which may contribute to differences in physical performance capacities such as sprinting, jumping, and change-of-direction ability [11, 12]. These factors could influence how players interact with task constraints and become involved in technical actions during SSGs. However, evidence for sex-related differences in technical skills is inconsistent, as limited differences were reported in several technical skill tests between male and female youth soccer players [13]. Furthermore, existing findings are primarily derived from invasion sports with different spatial, tactical, and physical demands than floorball. Consequently, it remains unclear whether age- or sex-related differences influence technical involvement during SSGs in youth floorball, where sport-specific evidence is currently lacking.

To date, no published studies have examined how SSG formats influence technical actions or performance indicators in floorball, despite its rapid growth as a stick-based team sport and its status as the largest indoor sport in Northern Europe. The sport involves five field players and a goaltender on a 40 × 20 m court and is characterized by distinct task constraints, including an indoor playing area, frequent substitutions, and the use of light sticks that demand rapid ball handling and precise technical control. These characteristics create a high-paced, interactive game environment that differs from many other invasion sports. As a result, findings from sports such as soccer, field hockey, and basketball cannot be readily generalized to floorball and may provide a limited or even misleading basis for training design. Taken together, there is a clear lack of sport-specific evidence on how SSG formats influence technical actions in youth floorball.

The primary aim of this study was to examine how different SSG formats influence technical actions by outfield players in floorball among children of various ages. A secondary aim was to explore whether these effects differ between age groups and whether they are impacted by biological sex. By providing evidence-based insights into how specific SSG formats affect player involvement across technical actions, the study aims to inform training design in youth floorball.

## Methods

### Participants

This observational study involved 24 floorball teams based in Stockholm County, Sweden. The teams comprised three boys’ teams and three girls’ teams across four age-based playing levels: 7–8 years, 10–11 years, 13–14 years, and 16–18 years. These age groups were chosen to align with the key developmental stages of the Swedish Floorball Development Model, encompassing movement enjoyment, skill development, purposeful training, and competitive play. These age groups, particularly the oldest group (16–18 years), may include variability in biological maturation despite similar chronological age. As biological maturity was not assessed, age group was considered a proxy for developmental stage. All athletes on each team were invited to participate, as the SSG session was conducted as part of regular training. No specific inclusion or exclusion criteria were applied.

Individual participant data were not collected due to the anonymized observational design, although each participating team typically consisted of approximately 15–25 players, resulting in an estimated total of approximately 360–600 participating athletes across the 24 teams. Participant characterization was therefore limited to team affiliation, sex category (boys’ or girls’ teams), and age-based playing level. Technical actions were recorded and analyzed at the team level, with repeated observations across SSG formats and repetitions forming the analytical dataset.

The study was submitted to the Swedish Ethical Review Authority for ethical approval before initiation, but the authority determined that formal ethical review was not required (decision Dnr 2022-03501-01). This decision was based on the following considerations: (1) no personal data would be collected, as the extracted data would be anonymous from video recordings, safeguarded by the Swedish Floorball Federation, and (2) the SSGs were part of standard training and did not involve any intervention or procedure requiring ethical review. Nevertheless, the study was conducted in accordance with the principles of the Declaration of Helsinki. This project was conducted in collaboration with the Swedish Floorball Federation, the Floorball Competence Center at Umeå University, and the Department of Community Medicine and Rehabilitation at Umeå University.

### Experimental design

The study adopted a nomothetic, follow-up, and multidimensional observational design [14], involving multiple teams, repeated observations across SSG formats and repetitions, and the simultaneous recording of several types of technical actions. The observational design followed an event-based approach, in which technical actions were operationalized as discrete events occurring within each SSG bout [2]. Each event was coded from video recordings using predefined operational definitions.

Four SSG formats representing typical floorball training configurations were used: 2v2 (20*10 m), 3v3 (20*12 m), 4v4 (20*20 m), and 5v5 (40*20 m, standard match play). Each format represents a combined manipulation of both player number and pitch size, reflecting realistic training conditions rather than isolated experimental factors. Consequently, the present design allows comparisons between SSG formats but does not allow causal conclusions regarding the separate effects of player number or pitch size.

Pitch sizes were selected to reflect ecologically valid floorball training environments, where changes in pitch dimensions and player numbers are inherently linked in practice. For example, a field with a standard 40*20 m pitch is often divided into two courts for 4v4 play, or four courts for 2v2 and 3v3 formats. All SSGs included goalkeepers and standard-sized goals (1.6 m wide, 1.15 m high). The SSG order was randomized in advance for each participating team to reduce various sources of error related to the SSG performance order.

All formats were played according to standard floorball rules regarding stick use, ball handling, goal scoring, and goalkeeper participation. No rule modifications were applied, and restarts following goals or out-of-play situations were performed immediately by the coach or nearest player to maintain game continuity and maximize effective playing time. Teams used their ordinary training equipment and played with standard floorball balls and sticks.

The SSGs were video recorded using one iPad (standard version) positioned on a tripod at the midpoint of the long side of each pitch, overlooking the entire field, and one iPhone (model 14) on a tripod placed in each corner of the opposite long side, directed diagonally toward the far half of the pitch. This setup provided excellent visibility of the entire pitch, thereby covering all outfield players and goalkeepers.

Data collection and data extraction from video footage occurred from spring 2023 to autumn 2024. Three raters independently assessed the footage using the same standardized coding protocol to provide more robust estimates of technical actions; one rater became unavailable and was replaced for approximately half of the footage. To ensure continuity in coding, the outgoing and incoming raters jointly reviewed a selection of video sequences to align their interpretation of the operational definitions, supporting inter-rater consistency across datasets. Prior to data collection, all raters participated in a training session in which the observation instrument and operational definitions were reviewed in detail. All raters had previous experience (several years) with floorball and familiarity with video-based performance analysis.

**Fig. 1:**
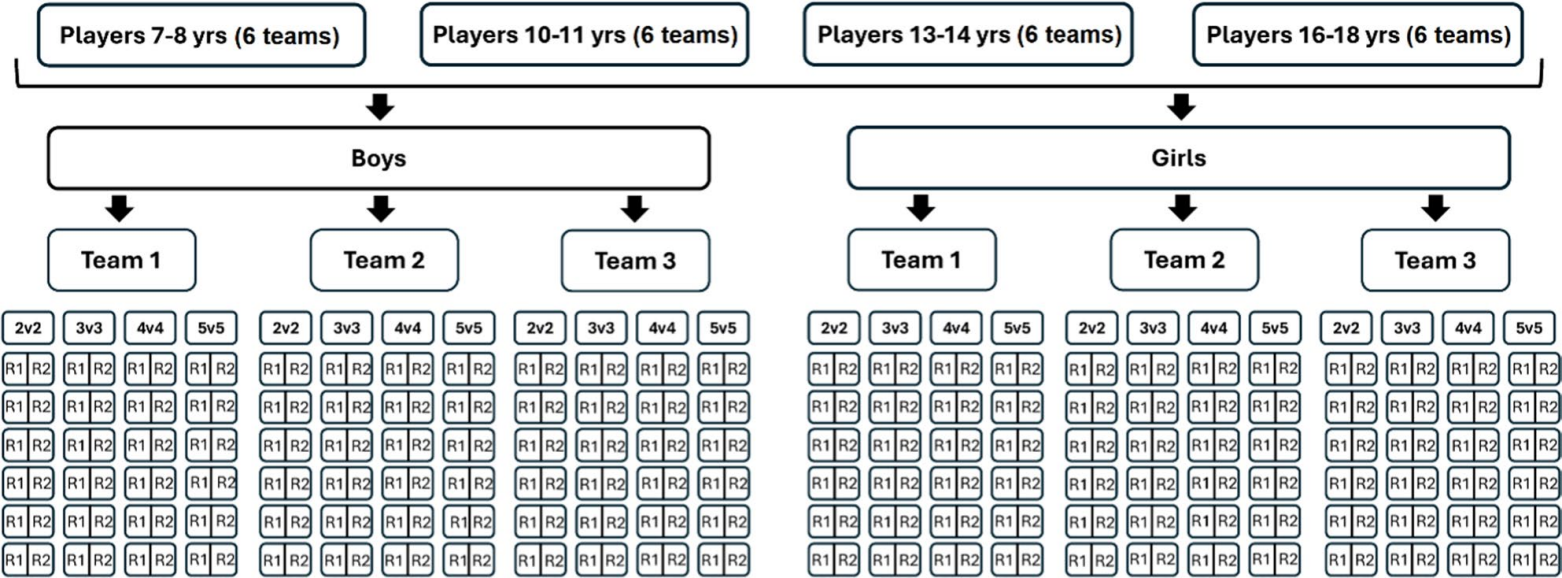
A schematic overview of the study design. The study involved 24 floorball teams, with six teams per age group (three boys’ teams and three girls’ teams). Each team completed small-sided games in 2v2, 3v3, 4v4, and 5v5 formats, performed in randomized order during a single training session. Each format was played six times, with 60 s bouts. All sessions were video recorded, and technical actions were independently assessed by two reviewers (R1 and R2), who evaluated the number of balls received, balls recovered, passes, and shots

All observations were collected during a single training session, minimizing variability associated with repeated testing. Each SSG lasted 60 s, which is considered reasonable given that shifts during a floorball match typically last around 30–50 s. Each SSG was repeated 6 times per team, with 6 teams per age group (3 male, 3 female), for a total of 36 repetitions per SSG within each age group. See Fig. 1 for a schematic overview of the study design.

Before each team’s training session began, players were divided into groups and were encouraged to warm up, so they were ready to start the games immediately. Information about the training content and the order of the game formats was provided to coaches and players in advance. The 60-minute training session was divided so that a maximum of 15 min was allocated to each SSG (four in total), including the setup of the pitch and the execution of six repetitions for each format.

### Technical actions

Technical actions by outfield players were coded as discrete events and analyzed both per player to describe individual involvement (e.g., total counts divided by the number of outfield players in the corresponding SSG format) and in total to describe the SSG format as a whole. The observation instrument was a structured category system specifically developed for this study and based on commonly used technical indicators in SSG research [5, 9, 10]. The instrument comprised four mutually exclusive and operationally predefined event categories (received balls, recovered balls, passes, and shots), and was therefore considered a low-openness observational system. Content validity was established through expert review by researchers with experience in floorball performance analysis and observational methodology. The raters independently coded each event from the video recordings and compiled the frequency counts for the following technical actions:

- Received balls: receiving a pass from a teammate.
- Recovered balls: gaining possession by physically winning the ball from an opponent, intercepting a pass, or gaining control of a loose ball of which no player has possession. These events were combined as a general indicator of regaining possession during play.
- Passes: intentional action where the ball is played to a teammate.
- Shots: intentional action where the player aims to shoot the ball toward the goal.

The composite outcome game volume was also calculated to provide an overall indicator of player involvement [15]:

- Game volume: (Received balls + Recovered balls) / number of players.

Therefore, 10 outcomes were analyzed in total (five per-player variables and five total-count variables). Each rater’s compiled dataset was sent to one of the authors, who compiled the complete dataset and sent it to another author for analysis. Inter-rater reliability was assessed for each technical action (except game volume) within each age group using Cronbach’s alpha, with coefficients ranging from 0.54 to 0.94 (see details in Table S1 in Supplementary Material).

### Statistical analysis

The average of both rater’s compiled count for each technical action per SSG repetition was used in the analyses to provide more robust estimates of the technical actions. The resulting dataset included 576 rows, representing team-level observations across the 24 Teams, each performing four SSG formats, each repeated six times.

The dataset structure reflected a repeated-measures design at the team level, with each team contributing repeated observations across SSG formats and repetitions. Two types of outcome variables were analyzed separately: (i) player-normalized outcomes, reflecting per-player involvement (i.e., technical actions divided by outfield players in each SSG format), and (ii) total team counts, reflecting absolute frequencies of technical actions within each SSG bout. The technical action outcomes were screened for normality using histograms and Q-Q plots. Although the outcomes represented event counts, their distributions showed sufficient variability and approximated normality; therefore, linear mixed models were considered appropriate for analysis.

Given the hierarchical nature of the data, with repeated observations nested within teams, separate two-level multilevel linear models (MLMs) were estimated for each outcome. Level 1 comprised repeated observations (within-team level), and Level 2 represented team membership (between-team level). Each model included a random intercept for team to account for clustering, and fixed effects for sex (male/female), age group (four categories), SSG format (four levels), and the interaction between age group*SSG. Post hoc pairwise comparisons with Bonferroni correction were applied to significant fixed effects within each MLM. When a significant age group*SSG interaction was observed, additional repeated ANOVAs were conducted to examine differences between SSG formats within each age group using Bonferroni-adjusted comparisons.

Models were estimated using maximum likelihood with a variance components covariance structure for the random intercept. Model performance was evaluated using type III tests of fixed effects. Model fit was further assessed using marginal R² (variance explained by fixed effects) and conditional R² (variance explained by both fixed and random effects), using pseudo-R² measures appropriate for MLMs. All analyses were conducted using IBM SPSS Statistics (v28; SPSS Inc., IBM, Chicago, IL, USA). Statistical significance was set at *p* < 0.05.

## Results

### Main effects from the multilevel linear models (MLMs)

Across all MLMs, SSG format was a significant predictor of player involvement for all outcomes. Age group had a significant main effect on balls received, passes, and game volume, but not on balls recovered or shots. Sex was not a significant predictor in any model. Significant interactions between age group*SSG were shown for balls received, balls recovered, passes, and game volume, but not shots. Model fit statistics for each technical action, including marginal and conditional R² values, are presented in Table 1.

**Table 1:** Summary of results for fixed effects for outcomes with counts per player (PP) and in total (Tot)

Outcome		Sex F(1,24)	Age group F(3,24)	SSG F(3,552)	Age group*SSG F(9,552)	Marg. *R*^2^	Cond. *R*^2^
Balls received	PP	F = 0.63 *p =* 0.44	F = 18.59 *p <* 0.001*	F = 113.95 *p <* 0.001*	F = 1.27 *p =* 0.25	0.48	0.54
	Tot	F = 0.86 *p =* 0.36	F = 21.12 *p <* 0.001*	F = 15.08 *p <* 0.001*	F = 3.17 *p <* 0.001*	0.37	0.46
Balls recovered	PP	F = 2.47 *p =* 0.13	F = 0.25 *p =* 0.86	F = 274.50 *p <* 0.001*	F = 4.00 *p <* 0.001*	0.59	0.61
	Tot	F = 2.11 *p =* 0.16	F = 0.43 *p =* 0.74	F = 15.64 *p <* 0.001*	F = 4.31 *p <* 0.001*	0.13	0.18
Passes	PP	F = 3.50 *p =* 0.07	F = 18.70 *p <* 0.001*	F = 83.62 *p <* 0.001*	F = 2.10 *p =* 0.03*	0.42	0.46
	Tot	F = 2.67 *p =* 0.12	F = 18.46 *p <* 0.001*	F = 85.01 *p <* 0.001*	F = 3.32 *p <* 0.001*	0.45	0.51
Shots	PP	F = 0.78 *p =* 0.39	F = 0.03 *p =* 0.99	F = 79.54 *p <* 0.001*	F = 0.41 *p =* 0.93	0.29	0.31
	Tot	F = 1.50 *p =* 0.23	F = 0.06 *p =* 0.98	F = 18.06 *p <* 0.001*	F = 0.44 *p =* 0.91	0.09	0.12
Game volume	PP	F = 0.13 *p =* 0.72	F = 20.39 *p <* 0.001*	F = 525.11 *p <* 0.001*	F = 4.25 *p <* 0.001*	0.74	0.76
	Tot	F = 0.01 *p =* 0.95	F = 21.22 *p <* 0.001*	F = 31.57 *p <* 0.001*	F = 1.56 *p =* 0.13	0.34	0.40

*Cond*. Conditional, *Marg*. Marginal, *SSG *small-sided game format

Asterisks indicate statistical significance at *p*< 0.05

### Post hoc analyses

#### Age group

Players aged 7-8 and 10-11 had significantly fewer counts of received balls, passes and game volume than players aged 13-14 and 16-18 across sexes and SSG formats, both per player and in total (p ≤ 0.007). No significant differences were observed between the two younger (7-8 vs 10-11) and older (13-14 vs 16-18) age groups for these outcomes (p = 0.37-1.0). See Table 2 for descriptive data on the technical actions divided by age group.

**Table 2:** Data* for outcomes for each age group with counts per player (PP) and in total (Tot) presented in mean, standard deviation (SD), and percentage to the 16–18 age group mean data (defined as 100%)

Outcome		7–8 yrs			10–11 yr			13–14 yr			16–18 yr	
		Mean	SD	%	Mean	SD	%	Mean	SD	%	Mean	SD
Balls received	PP	0.7	0.4	57	0.8	0.4	70	1.1	0.5	95	1.2	0.4
	Tot	4.4	1.7	55	5.4	2.0	68	7.3	2.2	92	7.9	2.8
Balls recovered	PP	1.2	0.4	101	1.2	0.5	102	1.2	0.5	104	1.1	0.5
	Tot	7.5	2.1	105	7.3	2.1	104	7.4	2.0	104	7.1	2.1
Passes	PP	1.2	0.5	72	1.3	0.5	80	1.7	0.5	101	1.6	0.5
	Tot	7.7	2.5	70	8.6	2.7	78	10.8	2.8	98	11.0	3.7
Shots	PP	0.3	0.2	100	0.3	0.2	101	0.3	0.2	103	0.3	0.2
	Tot	1.5	1.1	99	1.5	1.2	101	1.6	1.2	103	1.5	1.1
Game volume	PP	1.8	0.6	79	2.0	0.7	85	2.3	0.8	99	2.3	0.7
	Tot	11.8	2.5	79	12.7	2.4	85	14.6	2.3	98	15.0	2.7

*Data is collapsed across the 3 male and 3 female teams, 4 small-sided game formats, and 6 repetitions per small-sided game format, to match the post hoc analyses’ structure for age group

#### SSG format

Across all five outcomes, the 2v2 SSG format showed higher per-player values than all larger formats across sexes and age groups, and each step toward larger team sizes (3v3, 4v4, 5v5) was associated with lower per-player counts (*p <* 0.001 for all comparisons). For total counts, larger formats (especially 5v5) showed higher values for balls received and passes, but fewer shots, than smaller SSG formats. The 4v4 format showed higher game volume compared with all other SSG formats and more balls recovered than 2v2 and 5v5 (*p <* 0.001 for both). No differences between SSG formats were observed for balls received for 3v3 vs. 4v4 (*p =* 0.10) and 4v4 vs. 5v5 (*p* < 1.0), balls recovered between 2v2 vs. 5v5 (*p =* 1.0) and 3v3 vs. 4v4 (*p =* 0.56), passes between 4v4 vs. 5v5 (*p =* 1.0), shots between 2v2 vs. 3v3 (*p =* 1.0), 2v2 vs. 4v4 (*p =* 1.0), and 3v3 vs. 4v4 (*p =* 0.30), and game volume between 3v3 vs. 5v5 (*p =* 1.0). See Table 3 for descriptive data on the technical actions divided by SSG format.

**Table 3:** Data* for outcomes for each small-sided game format with counts per player (PP) and in total (Tot) presented in mean, standard deviation (SD), and percentage to the 5v5 mean data (defined as 100%)

Outcome		2v2			3v3			4v4			5v5	
		Mean	SD	%	Mean	SD	%	Mean	SD	%	Mean	SD
Balls received	PP	1.4	0.6	199	1.0	0.4	148	0.8	0.3	121	0.7	0.3
	Tot	5.4	2.3	79	6.0	2.4	89	6.6	2.7	97	6.8	2.9
Balls recovered	PP	1.7	0.5	246	1.3	0.3	186	1.0	0.3	146	0.7	0.2
	Tot	6.7	1.8	98	7.6	2.1	111	8.0	2.1	117	6.8	2.1
Passes	PP	1.8	0.6	162	1.6	0.5	145	1.3	0.3	123	1.1	0.3
	Tot	7.1	2.5	65	9.4	2.8	87	10.7	2.8	99	10.9	3.4
Shots	PP	0.4	0.3	435	0.3	0.2	309	0.2	0.1	201	0.1	0.1
	Tot	1.7	1.2	174	1.8	1.2	186	1.6	1.1	161	1.0	0.8
Game volume	PP	3.0	0.7	222	2.3	0.5	167	1.8	0.3	134	1.4	0.3
	Tot	12.2	2.7	89	13.7	2.8	100	14.6	2.5	107	13.7	2.7

*Data is collapsed across the 3 male and 3 female teams, 4 age groups, and 6 repetitions per small-sided game format, to match the post hoc analyses’ structure for SSG format

#### Interaction between age group and SSG format

Significant interactions between age group and SSG format were found for several outcomes. For balls received, the significant interaction was observed for total counts only, whereas for game volume, it was observed for per-player values only. Players aged 16–18 years showed lower total counts in the 2v2 format compared to 3v3 (*p =* 0.02), 4v4 (*p <* 0.001), and 5v5 (*p <* 0.001) formats, which were not observed for the younger age groups, apart from players 13–14 for 2v2 vs. 5v5 (*p <* 0.001). For balls recovered, players aged 7–8 years showed a larger increase in total counts from 2v2 to 3v3 (*p <* 0.001) and from 3v3 to 4v4 (*p =* 0.02) compared to the other age groups. In contrast, the 16–18 year age group showed a higher decrease in balls recovered per player than the other age groups from 3v3 to 4v4 (*p =* 0.01).

**Fig. 2:**
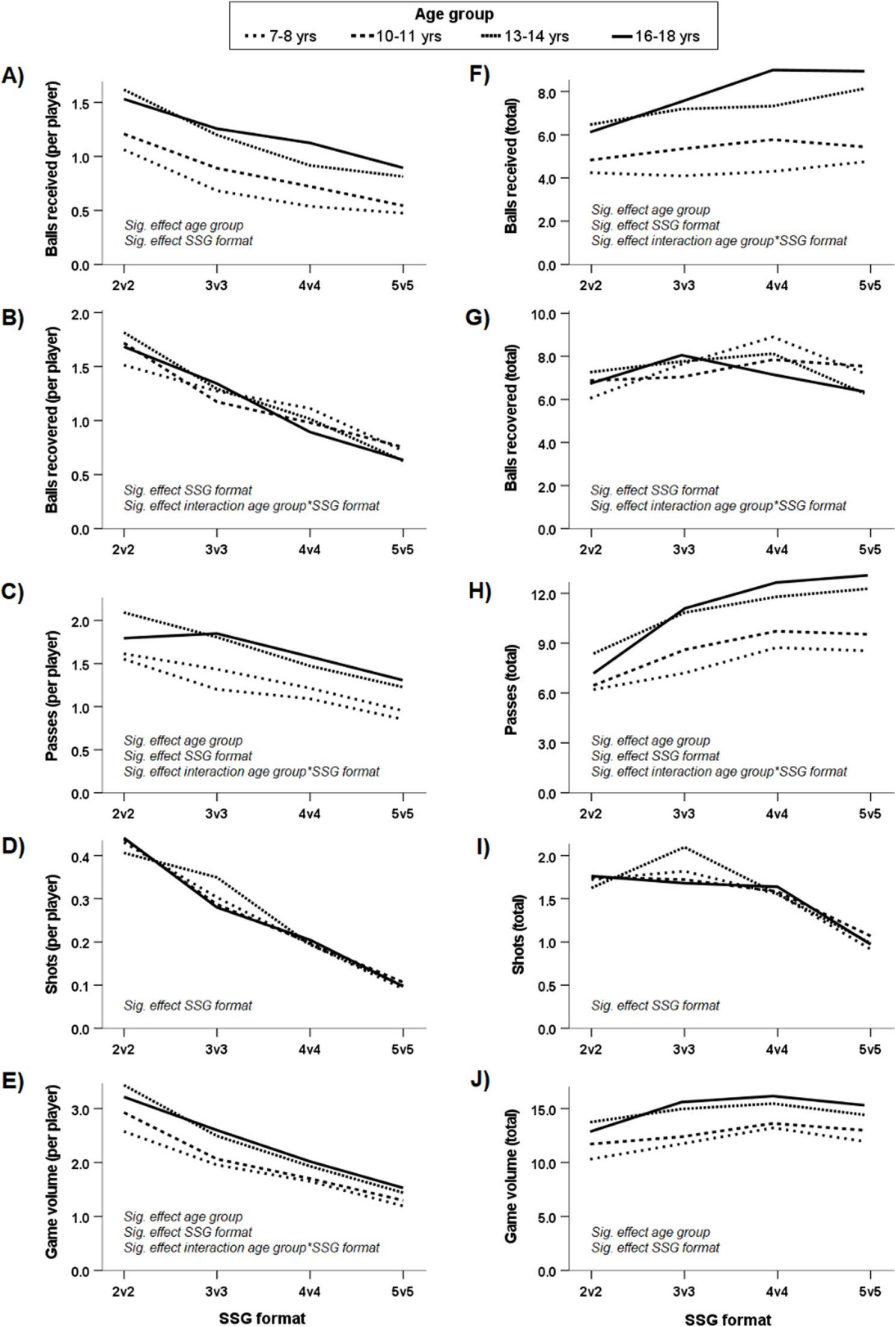
Mean data for the technical actions with counts per player (**A-E**) and in total (**F-J**) across the small-sided game (SSG) format for each age group

Passes in total counts increased from 2v2 to 3v3 in all age groups (*p <* 0.001 for all) except for players 7–8 years (*p =* 0.33) and was highest among players 16–18 years. Passes also increased from 3v3 to 5v5 in the two older age groups (*p =* 0.03 for both) but not the two younger age groups (*p =* 0.08–0.76). The increase in game volume per player from 4v4 to 3v3 was larger in the two older compared to younger age groups. See Fig. 2 for the mean technical action data for each SSG format per age group, which illustrates the direction and relative magnitude of the age group*SSG format interaction patterns. Full cross-tabulated values of technical actions divided by sex, age group, and SSG format are available in Tables S2-S3 in Supplementary Material.

## Discussion

This is the first study to investigate how different SSG formats affect technical actions among male and female youth floorball outfield players across age groups. The main findings show that the SSG format was associated with clear differences in technical involvement, with smaller formats leading to greater engagement per player. In contrast, the total number of technical actions varied more between SSGs. Age influenced several outcomes, whereas biological sex did not. Significant interactions between age group and SSG format for several outcomes indicate that players of different ages respond differently to specific SSGs.

Across all outcomes, the SSG format emerged as a consistent predictor of technical involvement, with smaller formats, particularly 2v2, resulting in markedly greater action counts per player. This finding aligns with research in other team sports demonstrating that smaller SSG formats with fewer players were associated with higher individual involvement, ball contacts, and decision-making opportunities [5, 9, 10]. Notably, these studies also manipulated player number and pitch size simultaneously, although they attempted to maintain a relatively consistent playing area per player across formats. From an ecological dynamics and nonlinear pedagogy perspective [6–8], smaller SSGs constrain the environment in ways that shape the affordances available to players, promoting more frequent perception-action couplings and greater functional variability in technical behavior. However, it is important to emphasize that player number and pitch size were manipulated jointly across formats; therefore, observed differences reflect combined task constraints rather than isolated effects of each variable. In contrast to previous studies [5, 9, 10], pitch dimensions in the present study were selected to reflect common floorball training practices and standard pitch subdivisions, prioritizing ecological validity over strictly controlling relative playing area per player.

A distinction emerged between per-player involvement and total technical actions for the SSGs. While each reduction in player numbers increased per-player involvement, larger formats often yielded higher total counts of balls received and passes, though this varied by age group (Fig. 2). Recovered balls should be interpreted as a general indicator of regaining possession, as this outcome included different forms of possession regain (e.g., interceptions, challenges, and loose-ball recoveries) that may involve distinct technical and tactical processes. Notably, total shot counts were substantially lower in the 5v5 format across all ages (in mean values: 1.0 vs. 1.6–1.8). Together, these findings suggest that different SSG formats may serve distinct developmental purposes. Smaller formats may be useful when the aim is to increase individual engagement and technical involvement, whereas larger formats may provide a context that more closely resembles aspects of full-game dynamics. However, interpretations regarding tactical development remain speculative, as tactical behaviors were not directly measured in the present study. In this context, this distinction aligns with principles of nonlinear pedagogy, emphasizing the purposeful manipulation of task constraints to elicit specific learning outcomes rather than reliance on a single “optimal” format [8].

Age group significantly influenced several technical outcomes, with older players generally receiving more balls, completing more passes, and exhibiting greater overall game volume than younger players. These differences may reflect age-related developments in physical capacity, perceptual skills, and tactical understanding, enabling older youths to exploit available space more effectively and sustain involvement for longer periods [9, 10]. As training is typically organized by chronological age, these findings have practical relevance for coaches designing age-appropriate SSGs.

In contrast, no significant main effects of biological sex were observed for any technical action, indicating no observed differences in levels of technical involvement between male and female youth players within the present SSG formats. Given floorball’s emphasis on quick stick handling, positioning, and rapid decision-making, physical differences between the sexes may be less influential in this context than in sports that place greater demands on strength or speed [11, 12]. Taken together, these findings suggest that similar SSG formats may be appropriate for male and female youth floorball players when the aim is to promote technical involvement. However, further research using complementary measures is needed to confirm this.

An important finding of this study is the identification of significant interactions between age group and SSG format, revealing that differences between SSG formats vary across age groups. For example, players aged 7–8 years showed larger relative increases in balls recovered than the two older groups when progressing from very small to moderately sized formats. Conversely, older players, particularly those aged 16–18 years, received more balls and made more passes, and had a higher total game volume in the 2v2 format than the two younger age groups. They also demonstrated a larger relative increase in total counts for these outcomes in the 2v2 format compared with the larger SSGs. Similar age-dependent responses to SSG formats have been reported in soccer, where larger formats appear to support technical involvement among more mature players better [10]. These findings highlight how players at different developmental stages respond differently to task constraints.

From a pedagogical perspective, the results suggest that SSG design may be aligned with players’ developmental characteristics. Smaller SSG formats may increase the frequency of technical involvements, while larger formats may provide different spatial and interactional conditions that are more representative of full-game situations. However, whether these differences translate into enhanced tactical behaviors remains to be established. Within a nonlinear pedagogical framework, task constraints should be sufficiently challenging to promote exploration without overly constraining functional solutions [6, 8]. Taken together, the findings suggest that the effectiveness of SSG design may depend on players’ age. Smaller formats appear particularly suitable for increasing younger players’ engagement, whereas progressively larger formats may better support tactical involvement among older youths.

These insights align with the Swedish Floorball Development Model and highlight SSG usage as a practical coaching tool for aligning training environments with age-specific objectives. Coaches may therefore consider adjusting SSG formats according to player’s developmental stage to manipulate technical involvement and training demands. Beyond performance development, SSGs offer scalable and engaging training formats that may support motivation and long-term participation in youth sport.

Although floorball research has historically been limited, recent years have seen increased attention to, e.g., motor control and performance [16, 17], injury epidemiology [18–20], and injury prevention [21]. The present study extends this literature by providing sport-specific evidence on how SSG format affects technical actions in youth floorball.

### Strengths and limitations

A strength was the large sample of 24 teams comprising three boys’ teams and three girls’ teams per age group, which is larger than in most studies focusing on SSGs. Another strength is the randomization of SSG order in advance for each participating team, which improves data quality by reducing various sources of error related to SSG performance order.

Nevertheless, several limitations related to the study design and data collection procedures should be acknowledged. (i) All data were collected during a single training session, which limits conclusions about longer-term adaptations. (ii) SSG durations were relatively short (60 s) to reflect typical match shift durations in floorball, which likely favored high-intensity, short-duration technical actions while underrepresenting longer, more organized phases of play. (iii)

Inter-rater reliability varied across outcomes and age groups, with lower agreement observed particularly for balls recovered and among younger players. Although multiple independent raters and averaged counts were used to reduce random measurement error, some measurement uncertainty remains.

Additional limitations related to the scope of the outcome measures and the interpretation of the observed SSG effects. (iv) The study focused on frequency-based technical actions and did not include qualitative or tactical/positional measures, limiting the ability to assess performance quality and broader game dynamics. (v) Chronological age was used as a proxy for development because anonymized data collection precluded assessment of biological maturation and other individual developmental characteristics. (vi) Field dimensions were selected to reflect practical and ecological considerations of floorball, such that differences across SSG formats represent combined task constraints rather than isolated variables. (vii) the multilevel modeling accounted for team-level clustering, as player-level longitudinal tracking was not possible due to the study design. Finally, (viii) teams were recruited from Stockholm County only, a large a diverse county including both urban and suburban municipalities, which may limit the generalizability of findings to other geographical regions or floorball contexts.

### Future research

Future research should examine how technical actions interact with tactical behaviors and physical load across SSG formats by incorporating more comprehensive indicators, such as positional play, decision-making quality, and measures of biological maturation. Such work could provide a more complete understanding of player behavior and developmental differences in youth floorball.

## Conclusions

This study demonstrates that smaller SSG formats were associated with higher individual involvement across all technical actions, whereas total technical-action counts varied more between formats. The influence of SSG format differed across age groups, indicating that players of different ages responded differently to the game configurations, while no differences related to biological sex were observed. These findings indicate that age should be considered when selecting SSG formats in youth floorball. From a practical perspective, coaches can use smaller formats to increase technical involvement and engagement, particularly among younger players. Overall, the results support the purposeful use of SSGs as a flexible training tool for aligning practice environments with age-specific developmental objectives.

## Supplementary Information

Supplementary Material 1.

## Data Availability

The datasets used and analyzed during the current study are available from the corresponding author on reasonable request.

## Abbreviations

MLM: Multilevel linear model
SSG: Small-sided game

## References

[1] Smith W. Fundamental movement skills and fundamental games skills are complementary pairs and should be taught in complementary ways at all stages of skill development. Sport Educ Soc. 2016;21:431–42. 10.1080/13573322.2014.927757.

[2] Clemente FM, Afonso J, Sarmento H. Small-sided games: An umbrella review of systematic reviews and meta-analyses. PLoS ONE. 2021;16:e0247067. 10.1371/journal.pone.0247067. 33577611 10.1371/journal.pone.0247067 PMC7880470

[3] Halouani J, Chtourou H, Gabbett T, Chaouachi A, Chamari K. Small-sided games in team sports training: a brief review. J Strength Cond Res. 2014;28:3594–618. 10.1519/JSC.0000000000000564. 24918302 10.1519/JSC.0000000000000564

[4] Pizarro D, Práxedes A, Travassos B, Moreno A. Development of Defensive Actions in Small-Sided and Conditioned Games With Offensive Purposes in Futsal. Front Psychol. 2020;11:591572. 10.3389/fpsyg.2020.591572. 33192934 10.3389/fpsyg.2020.591572 PMC7649284

[5] Timmerman EA, Savelsbergh GJP, Farrow D. Creating Appropriate Training Environments to Improve Technical, Decision-Making, and Physical Skills in Field Hockey. Res Q Exerc Sport. 2019;90:180–9. 10.1080/02701367.2019.1571678. 30794115 10.1080/02701367.2019.1571678

[6] Chow JY, Davids K, Button C, Shuttleworth R, Renshaw I, Araújo D. The Role of Nonlinear Pedagogy in Physical Education. Rev Educ Res. 2007;77:251–78. 10.3102/003465430305615.

[7] Zelaznik HN. The Past and Future of Motor Learning and Control: What Is the Proper Level of Description and Analysis? Kinesiol Rev. 2014;3:38–43. 10.1123/kr.2014-0035.

[8] Tan CWK, Yi CJ, Davids K. How does TGfU work?’: examining the relationship between learning design in TGfU and a nonlinear pedagogy. Phys Educ Sport Pedagogy. 2012;17:331–48. 10.1080/17408989.2011.582486.

[9] Clemente FM, Sanches R, Moleiro CF, Gomes M, Lima R. Technical Performance and Perceived Exertion Variations Between Small-Sided Basketball Games in Under-14 and Under-16 Competitive Levels. J Hum Kinet. 2020;71:179–89. 10.2478/hukin-2019-0082. 32148582 10.2478/hukin-2019-0082 PMC7052724

[10] King M, Barrett S, McLellan R, Cox J, Brown M, Mackenzie S, Towlson C. Does team size affect Scottish male academy soccer player technical, locomotor and psychosocial outcomes during age and maturity bio-banded small-sided games? J Sports Sci. 2025;1:1–11. 10.1080/02640414.2025.2456408.Epub ahead of print. 10.1080/02640414.2025.2456408 39905683

[11] Atkinson MA, James JJ, Quinn ME, Senefeld JW, Hunter SK. Sex Differences in Track and Field Elite Youth. Med Sci Sports Exerc. 2024;56:1390–7. 10.1249/MSS.0000000000003423. 38595163 10.1249/MSS.0000000000003423

[12] Tingelstad LM, Raastad T, Myklebust G, Gjerstad Andersen TE, Solstad BE, Bugten JB, Luteberget LS. Age and Sex Differences in Physical Performance Among Adolescent Team Sport Athletes. Eur J Sport Sci. 2025;25:e12284. 10.1002/ejsc.12284. 40205849 10.1002/ejsc.12284 PMC11982625

[13] Sørensen A, Haugen EC, van den Tillaar R. Is There a Sex Difference in Technical Skills among Youth Soccer Players in Norway? Sports. 2022;10:50. 10.3390/sports10040050. 35447860 10.3390/sports10040050 PMC9027718

[14] von Fircks E. From Nomothetic and Idiographic to Synthography. Integr Psychol Behav Sci. 2025;59:89. 10.1007/s12124-025-09944-1. 41324849 10.1007/s12124-025-09944-1

[15] Grehaigne J-F, Godbout P. Performance Assessment in Team Sports. J Teach Phys Educ. 1997;16:500–16. 10.1123/jtpe.16.4.500.

[16] Farana R, Brtva P, Irwin G, Hamill J. Head-Trunk Coordination During Shooting Skills in Young Floorball Players. Res Q Exerc Sport. 2025;96:555–62. 10.1080/02701367.2025.2461321. 40019791 10.1080/02701367.2025.2461321

[17] Vecbērza L, Šmite Z, Plakane L, Ābeļkalns I. The Impact of Ankle Plantar-Flexor Muscle Strength on Sprint Acceleration in Floorball Players. Int J Sports Physiol Perform. 2025;20:393–8. 10.1123/ijspp.2024-0272. 39837318 10.1123/ijspp.2024-0272

[18] Levin S, Tervo T, Ivarsson A, Hägglund M, Stenling A. Combinations of psychological and physical risk factors for sport injuries in youth floorball players: a latent profile analysis. BMJ Open Sport Exerc Med. 2025;11:e002309. 10.1136/bmjsem-2024-002309. 40129478 10.1136/bmjsem-2024-002309 PMC11931955

[19] Olli K, Mari L, Pekka K, Kathrin S, Tommi V, Tanja K, et al. Postural Control as a Risk Factor for Noncontact Anterior Cruciate Ligament Injury in Youth Female Basketball and Floorball Athletes. Scand J Med Sci Sports. 2025;35:e70081. 10.1111/sms.70081. 40442947 10.1111/sms.70081 PMC12123058

[20] Pasanen K, Parkkari J, Äyrämö S, Vasankari T, Krosshaug T, Leppänen M. No association between knee biomechanics during 90-degree cutting maneuver and future anterior cruciate ligament injury in female floorball players. Clin Biomech. 2025;126:106571. 10.1016/j.clinbiomech.2025.106571. 10.1016/j.clinbiomech.2025.106571 40460753

[21] Åkerlund I, Sonesson S, Lindblom H, Hagelin E, Carlfjord S, Hägglund M. I’d rather do that (Knee Control) than be injured and not able to play: a qualitative study on youth floorball players’ and coaches’ perspectives of how to overcome barriers for injury prevention exercise programme use. BMJ Open Sport Exerc Med. 2024;10:e001953. 10.1136/bmjsem-2024-001953. 39224202 10.1136/bmjsem-2024-001953 PMC11367341

