# Supplementary material for: Small-sided games in floorball: impact on technical actions in players aged 7–18
Source: BMC Sports Sci Med Rehabil. 2026 Aug 15;18:360. doi: 10.1186/s13102-026-01992-3 (PMC13483565; doi:10.1186/s13102-026-01992-3)
Supplement: Supplementary file 1 [file 13102_2026_1992_MOESM1_ESM.docx]

**SUPPLEMENTARY MATERIAL**

**Inter-rater reliability**

The few technical actions with lower reliability (Chronbach’s α < 0.6) involved children aged 7-8 years for balls received and balls recovered (Chronbach’s α = 0.54-0.55), and balls recovered for children 13-14. Overall, it seems that the youngest age group 7-8 years and the technical action balls recovered were more difficult to accurately judge.

**Table S1**. Inter-rater reliability for all outcomes collapsed across sex, divided across age groups, presented with Cronbach’s alpha. (values <0.6 in red, 0.6-0.79 in black, ≥0.8 in bold).

| *Outcome* | *Age group* | *Cronbach’s α* |
| --- | --- | --- |
| Balls received | 7-8 yrs | 0.55 |
|  | **10-11 yrs** | **0.84** |
|  | 13-14 yrs | 0.68 |
|  | **16-18 yrs** | **0.86** |
| Balls recovered | 7-8 yrs | 0.60 |
|  | 10-11 yrs | 0.73 |
|  | 13-14 yrs | 0.54 |
|  | 16-18 yrs | 0.68 |
| Passes | 7-8 yrs | 0.58 |
|  | **10-11 yrs** | **0.93** |
|  | 13-14 yrs | 0.76 |
|  | **16-18 yrs** | **0.90** |
| Shots | 7-8 yrs | 0.74 |
|  | **10-11 yrs** | **0.87** |
|  | 13-14 yrs | 0.74 |
|  | 16-18 yrs | 0.79 |

**Descriptive data for technical actions: counts per player**

**Table S2**. Data* for outcomes with counts per player presented in mean, standard deviation (SD), and percentage to the 5v5 mean data (defined to 100%).

| *Outcome* | *Age group* | *Sex* | *2v2* | | | *3v3* | | | *4v4* | | | *5v5 [ref]* | |
| --- | --- | --- | --- | --- | --- | --- | --- | --- | --- | --- | --- | --- | --- |
|  |  |  | Mean | SD | % | Mean | SD | % | Mean | SD | % | Mean | SD |
| Balls  received  *(per player)* | 7-8 yrs | M | 1.1 | 0.5 | 214 | 0.6 | 0.3 | 128 | 0.5 | 0.2 | 107 | 0.5 | 0.2 |
|  |  | F | 1.1 | 0.5 | 235 | 0.7 | 0.3 | 162 | 0.5 | 0.2 | 120 | 0.5 | 0.1 |
|  | 10-11 yrs | M | 1.4 | 0.4 | 215 | 1.0 | 0.3 | 161 | 0.8 | 0.2 | 126 | 0.6 | 0.3 |
|  |  | F | 1.0 | 0.5 | 231 | 0.8 | 0.3 | 168 | 0.6 | 0.2 | 142 | 0.5 | 0.2 |
|  | 13-14 yrs | M | 1.5 | 0.5 | 205 | 1.3 | 0.3 | 167 | 0.9 | 0.2 | 124 | 0.8 | 0.1 |
|  |  | F | 1.7 | 0.7 | 194 | 1.1 | 0.4 | 131 | 0.9 | 0.4 | 103 | 0.9 | 0.2 |
|  | 16-18 yrs | M | 1.4 | 0.6 | 160 | 1.3 | 0.3 | 139 | 1.2 | 0.4 | 128 | 0.9 | 0.4 |
|  |  | F | 1.6 | 0.4 | 183 | 1.3 | 0.4 | 143 | 1.1 | 0.3 | 123 | 0.9 | 0.3 |
| Balls  recovered  *(per player)* | 7-8 yrs | M | 1.6 | 0.5 | 230 | 1.2 | 0.3 | 169 | 1.1 | 0.2 | 157 | 0.7 | 0.2 |
|  |  | F | 1.4 | 0.4 | 191 | 1.4 | 0.4 | 183 | 1.1 | 0.2 | 152 | 0.7 | 0.2 |
|  | 10-11 yrs | M | 1.7 | 0.4 | 239 | 1.2 | 0.4 | 167 | 1.0 | 0.3 | 139 | 0.7 | 0.2 |
|  |  | F | 1.7 | 0.4 | 218 | 1.2 | 0.4 | 145 | 1.0 | 0.3 | 122 | 0.8 | 0.2 |
|  | 13-14 yrs | M | 1.5 | 0.4 | 231 | 1.1 | 0.2 | 169 | 1.0 | 0.2 | 146 | 0.7 | 0.2 |
|  |  | F | 2.1 | 0.4 | 355 | 1.5 | 0.3 | 248 | 1.1 | 0.2 | 179 | 0.6 | 0.2 |
|  | 16-18 yrs | M | 1.7 | 0.5 | 255 | 1.3 | 0.3 | 187 | 0.9 | 0.2 | 130 | 0.7 | 0.2 |
|  |  | F | 1.6 | 0.5 | 276 | 1.4 | 0.4 | 239 | 0.9 | 0.2 | 153 | 0.6 | 0.2 |
| Passes  *(per player)* | 7-8 yrs | M | 1.6 | 0.7 | 194 | 1.0 | 0.4 | 122 | 1.1 | 0.2 | 129 | 0.8 | 0.2 |
|  |  | F | 1.5 | 0.5 | 170 | 1.4 | 0.3 | 158 | 1.1 | 0.3 | 127 | 0.9 | 0.3 |
|  | 10-11 yrs | M | 1.7 | 0.6 | 160 | 1.5 | 0.3 | 145 | 1.2 | 0.2 | 120 | 1.0 | 0.3 |
|  |  | F | 1.6 | 0.5 | 180 | 1.4 | 0.4 | 157 | 1.2 | 0.3 | 136 | 0.9 | 0.2 |
|  | 13-14 yrs | M | 1.9 | 0.5 | 170 | 1.7 | 0.4 | 154 | 1.4 | 0.3 | 129 | 1.1 | 0.2 |
|  |  | F | 2.3 | 0.6 | 171 | 1.9 | 0.3 | 142 | 1.5 | 0.3 | 113 | 1.4 | 0.3 |
|  | 16-18 yrs | M | 1.7 | 0.5 | 130 | 1.7 | 0.4 | 133 | 1.5 | 0.4 | 121 | 1.3 | 0.4 |
|  |  | F | 1.9 | 0.7 | 144 | 2.0 | 0.4 | 149 | 1.6 | 0.3 | 120 | 1.3 | 0.4 |
| Shots  *(per player)* | 7-8 yrs | M | 0.5 | 0.3 | 500 | 0.4 | 0.1 | 356 | 0.2 | 0.1 | 229 | 0.1 | 0.1 |
|  |  | F | 0.4 | 0.2 | 433 | 0.3 | 0.3 | 300 | 0.2 | 0.1 | 192 | 0.1 | 0.1 |
|  | 10-11 yrs | M | 0.4 | 0.3 | 337 | 0.3 | 0.2 | 225 | 0.2 | 0.1 | 158 | 0.1 | 0.1 |
|  |  | F | 0.4 | 0.3 | 516 | 0.3 | 0.2 | 333 | 0.2 | 0.2 | 226 | 0.1 | 0.1 |
|  | 13-14 yrs | M | 0.3 | 0.2 | 352 | 0.3 | 0.3 | 370 | 0.2 | 0.1 | 207 | 0.1 | 0.1 |
|  |  | F | 0.5 | 0.3 | 462 | 0.4 | 0.2 | 342 | 0.2 | 0.2 | 189 | 0.1 | 0.1 |
|  | 16-18 yrs | M | 0.5 | 0.3 | 406 | 0.3 | 0.1 | 288 | 0.2 | 0.1 | 206 | 0.1 | 0.1 |
|  |  | F | 0.4 | 0.4 | 517 | 0.2 | 0.2 | 289 | 0.2 | 0.1 | 217 | 0.1 | 0.1 |
| Game volume  *(per player)* | 7-8 yrs | M | 2.7 | 0.6 | 223 | 1.8 | 0.3 | 152 | 1.6 | 0.2 | 136 | 1.2 | 0.2 |
|  |  | F | 2.5 | 0.7 | 208 | 2.1 | 0.4 | 175 | 1.7 | 0.3 | 140 | 1.2 | 0.3 |
|  | 10-11 yrs | M | 3.1 | 0.5 | 228 | 2.2 | 0.3 | 164 | 1.8 | 0.2 | 133 | 1.4 | 0.3 |
|  |  | F | 2.8 | 0.5 | 223 | 1.9 | 0.4 | 153 | 1.6 | 0.4 | 129 | 1.2 | 0.2 |
|  | 13-14 yrs | M | 3.1 | 0.4 | 217 | 2.4 | 0.3 | 168 | 1.9 | 0.2 | 135 | 1.4 | 0.2 |
|  |  | F | 3.8 | 0.5 | 259 | 2.6 | 0.2 | 178 | 2.0 | 0.3 | 134 | 1.5 | 0.3 |
|  | 16-18 yrs | M | 3.2 | 0.7 | 201 | 2.5 | 0.4 | 159 | 2.0 | 0.3 | 129 | 1.6 | 0.3 |
|  |  | F | 3.3 | 0.5 | 220 | 2.7 | 0.4 | 181 | 2.0 | 0.2 | 135 | 1.5 | 0.3 |

*Data is collapsed across each male (M) and female (F) grouping for each age group and small-sided game (3 teams*6 repetitions).

**Descriptive data for technical actions: counts in total**

**Table S3**. Data* for outcomes with counts in total presented in mean, standard deviation (SD), and percentage to the 5v5 mean data (defined to 100%).

| *Outcome* | *Age group* | *Sex* | *2v2* | | | *3v3* | | | *4v4* | | | *5v5 [ref]* | |
| --- | --- | --- | --- | --- | --- | --- | --- | --- | --- | --- | --- | --- | --- |
|  |  |  | Mean | SD | % | Mean | SD | % | Mean | SD | % | Mean | SD |
| Balls  received  *(in total)* | 7-8 yrs | M | 4.3 | 2.0 | 86 | 3.8 | 1.8 | 77 | 4.3 | 1.4 | 86 | 5.0 | 1.6 |
|  |  | F | 4.2 | 1.9 | 94 | 4.4 | 1.7 | 97 | 4.3 | 2.0 | 96 | 4.5 | 1.4 |
|  | 10-11 yrs | M | 5.5 | 1.7 | 86 | 6.2 | 1.8 | 97 | 6.4 | 1.7 | 101 | 6.4 | 2.5 |
|  |  | F | 4.2 | 2.1 | 93 | 4.5 | 1.7 | 101 | 5.1 | 1.4 | 114 | 4.5 | 1.7 |
|  | 13-14 yrs | M | 6.2 | 2.0 | 82 | 7.5 | 1.9 | 100 | 7.5 | 1.6 | 99 | 7.5 | 1.2 |
|  |  | F | 6.8 | 2.6 | 77 | 6.9 | 2.2 | 78 | 7.2 | 2.9 | 82 | 8.8 | 1.9 |
|  | 16-18 yrs | M | 5.8 | 2.3 | 64 | 7.5 | 2.0 | 83 | 9.3 | 2.8 | 102 | 9.1 | 3.7 |
|  |  | F | 6.5 | 1.7 | 73 | 7.6 | 2.3 | 86 | 8.7 | 2.2 | 99 | 8.8 | 3.2 |
| Balls  recovered  *(in total)* | 7-8 yrs | M | 6.4 | 1.9 | 92 | 7.1 | 1.7 | 102 | 8.8 | 1.7 | 125 | 7.0 | 1.6 |
|  |  | F | 5.7 | 1.5 | 76 | 8.2 | 2.3 | 110 | 9.1 | 2.0 | 122 | 7.4 | 2.2 |
|  | 10-11 yrs | M | 6.8 | 1.6 | 96 | 7.1 | 2.2 | 100 | 7.9 | 2.3 | 111 | 7.1 | 2.1 |
|  |  | F | 6.9 | 1.6 | 87 | 6.9 | 2.2 | 87 | 7.8 | 2.5 | 98 | 8.0 | 1.9 |
|  | 13-14 yrs | M | 6.2 | 1.4 | 93 | 6.8 | 1.3 | 101 | 7.8 | 1.9 | 117 | 6.7 | 1.8 |
|  |  | F | 8.4 | 1.5 | 142 | 8.8 | 1.8 | 149 | 8.4 | 1.9 | 143 | 5.9 | 2.1 |
|  | 16-18 yrs | M | 6.9 | 2.0 | 102 | 7.6 | 1.9 | 112 | 7.1 | 1.9 | 104 | 6.8 | 2.4 |
|  |  | F | 6.5 | 1.9 | 110 | 8.5 | 2.3 | 143 | 7.3 | 1.6 | 123 | 5.9 | 1.7 |
| Passes  *(in total)* | 7-8 yrs | M | 6.5 | 2.8 | 78 | 6.1 | 2.2 | 73 | 8.6 | 1.8 | 103 | 8.4 | 1.6 |
|  |  | F | 5.9 | 2.2 | 68 | 8.3 | 2.1 | 95 | 8.8 | 2.2 | 101 | 8.7 | 2.9 |
|  | 10-11 yrs | M | 6.6 | 2.4 | 64 | 9.0 | 1.8 | 87 | 10.0 | 1.8 | 96 | 10.4 | 3.0 |
|  |  | F | 6.3 | 2.1 | 72 | 8.2 | 2.3 | 94 | 9.5 | 2.7 | 109 | 8.7 | 2.3 |
|  | 13-14 yrs | M | 7.4 | 1.8 | 68 | 10.1 | 2.5 | 92 | 11.3 | 2.1 | 103 | 10.9 | 1.6 |
|  |  | F | 9.3 | 2.6 | 68 | 11.6 | 1.7 | 85 | 12.3 | 2.5 | 90 | 13.6 | 2.9 |
|  | 16-18 yrs | M | 6.6 | 2.0 | 52 | 10.2 | 2.2 | 80 | 12.4 | 3.3 | 97 | 12.7 | 3.7 |
|  |  | F | 7.7 | 2.7 | 57 | 12.0 | 2.6 | 89 | 12.9 | 2.0 | 96 | 13.4 | 3.7 |
| Shots  *(in total)* | 7-8 yrs | M | 2.0 | 1.2 | 200 | 2.1 | 0.8 | 214 | 1.8 | 0.9 | 183 | 1.0 | 1.1 |
|  |  | F | 1.4 | 1.0 | 173 | 1.5 | 1.5 | 180 | 1.3 | 1.1 | 153 | 0.8 | 0.8 |
|  | 10-11 yrs | M | 1.7 | 1.4 | 135 | 1.7 | 1.1 | 135 | 1.6 | 1.1 | 126 | 1.3 | 0.8 |
|  |  | F | 1.8 | 1.1 | 206 | 1.7 | 1.2 | 200 | 1.6 | 1.5 | 181 | 0.9 | 0.9 |
|  | 13-14 yrs | M | 1.3 | 0.8 | 141 | 2.0 | 1.6 | 222 | 1.5 | 0.7 | 166 | 0.9 | 0.6 |
|  |  | F | 2.0 | 1.3 | 185 | 2.2 | 1.0 | 205 | 1.6 | 1.3 | 151 | 1.1 | 1.0 |
|  | 16-18 yrs | M | 1.8 | 1.1 | 163 | 1.9 | 0.7 | 173 | 1.8 | 1.1 | 165 | 1.1 | 0.9 |
|  |  | F | 1.7 | 1.4 | 207 | 1.4 | 1.1 | 173 | 1.4 | 0.9 | 173 | 0.8 | 0.6 |
| Game volume  *(in total)* | 7-8 yrs | M | 10.7 | 2.3 | 89 | 10.9 | 1.8 | 91 | 13.0 | 1.3 | 109 | 12.0 | 1.7 |
|  |  | F | 9.9 | 2.8 | 83 | 12.6 | 2.6 | 105 | 13.4 | 2.4 | 112 | 11.9 | 2.8 |
|  | 10-11 yrs | M | 12.3 | 2.1 | 91 | 13.3 | 1.9 | 99 | 14.3 | 1.7 | 106 | 13.5 | 2.6 |
|  |  | F | 11.1 | 2.0 | 89 | 11.5 | 2.7 | 92 | 12.9 | 2.9 | 104 | 12.5 | 1.8 |
|  | 13-14 yrs | M | 12.3 | 1.7 | 87 | 14.3 | 2.1 | 101 | 15.3 | 2.0 | 108 | 14.2 | 1.9 |
|  |  | F | 15.1 | 2.1 | 103 | 15.6 | 1.4 | 107 | 15.6 | 2.5 | 107 | 14.6 | 2.6 |
|  | 16-18 yrs | M | 12.7 | 2.7 | 80 | 15.2 | 2.2 | 96 | 16.3 | 2.0 | 103 | 15.9 | 2.8 |
|  |  | F | 13.0 | 1.9 | 88 | 16.0 | 2.5 | 109 | 16.0 | 1.9 | 108 | 14.8 | 3.1 |

*Data is collapsed across each male (M) and female (F) grouping for each age group and small-sided game (3 teams*6 repetitions).
